# Subsequent Acupuncture Reverses the Aftereffects of Intermittent Theta-Burst Stimulation

**DOI:** 10.3389/fncir.2021.675365

**Published:** 2021-04-28

**Authors:** Xiao-Kuo He, Hui-Hua Liu, Shan-Jia Chen, Qian-Qian Sun, Guo Yu, Lei Lei, Zhen-Yuan Niu, Li-Dian Chen, Tsung-Hsun Hsieh

**Affiliations:** ^1^Fifth Hospital of XiaMen, Xiamen, China; ^2^Fujian University of Traditional Chinese Medicine, Fuzhou, China; ^3^Sun Yat-sen Memorial Hospital, Sun Yat-sen University, Guangzhou, China; ^4^School of Physical Therapy, Graduate Institute of Rehabilitation Science, Chang Gung University, Taoyuan, Taiwan; ^5^Neuroscience Research Center, Chang Gung Memorial Hospital, Taoyuan, Taiwan; ^6^Healthy Aging Research Center, Chang Gung University, Taoyuan, Taiwan

**Keywords:** acupuncture, cortical excitably, long-term potentiation, acquisition of learned skills, sequential visual isometric index finger abduction task

## Abstract

**Objective:**

This study explored whether acupuncture affects the maintenance of long-term potentiation (LTP)-like plasticity induced by transcranial magnetic stimulation (TMS) and the acquisition of motor skills following repetitive sequential visual isometric pinch task (SVIPT) training.

**Methods:**

Thirty-six participants were recruited. The changes in the aftereffects induced by intermittent theta-burst stimulation (iTBS) and followed acupuncture were tested by the amplitude motor evoked potential (MEP) at pre-and-post-iTBS for 30 min and at acupuncture-in and -off for 30 min. Secondly, the effects of acupuncture on SVIPT movement in inducing error rate and learning skill index were tested.

**Results:**

Following one session of iTBS, the MEP amplitude was increased and maintained at a high level for 30 min. The facilitation of MEP was gradually decreased to the baseline level during acupuncture-in and did not return to a high level after needle extraction. The SVIPT-acupuncture group had a lower learning skill index than those in the SVIPT group, indicating that acupuncture intervention after SVIPT training may restrain the acquisition ability of one’s learning skills.

**Conclusion:**

Acupuncture could reverse the LTP-like plasticity of the contralateral motor cortex induced by iTBS. Subsequent acupuncture may negatively affect the efficacy of the acquisition of learned skills in repetitive exercise training.

## Introduction

Motor learning is the process of acquiring motor skills in repetitive training, in which the execution of actions is improved by goal-oriented training (Roth et al., 2020). When given a series of varying motor learning items, subsequent movements may interfere with the imprinted memory stimulated by previous motor learning (Hulme et al., 2013). In order to learn the different motor skills in sequential order, the acquired memory for the previous motor skill would be inhibited by the later movement (Hulme et al., 2013). Several preclinical studies have found that skill learning in the previous movement can be influenced by subsequent behavioral activity. For example, in a rat model, when stimulated by differing frequencies of electric stimulation, the hippocampal trisynaptic loop demonstrated the efficiency of frequency-dependent synaptic transmission, known as long-term potentiation (LTP) and long-term depression (LTD)-like plasticity (Huang and Kandel, 2005). Several studies have supported the hypothesis that LTP is critical in memory formation and consolidation (Kramar and Lynch, 2003). The presence of LTP could be reversed by subsequent electrical stimulation or depotentiation. This was demonstrated by Dong et al., 2020, who applied low-frequency electrical stimulation and found a form-and time-dependent effect on the maintenance of LTP of the hippocampus (Dong et al., 2020). Similarly, LTP of hippocampal synapses in adult rats was reversed as rats entered a novel environment (Xu et al., 1998).

Acupuncture is a traditional Chinese therapeutic method and is widely used in clinical practice to treat diseases such as hemiplegia, stroke, and Alzheimer’s disease (Wang et al., 2018; Zheng et al., 2018). Previous research has suggested that electroacupuncture could restore hippocampal LTP in rats (He et al., 2012). Clinical data also indicated that acupuncture could modulate corticomotoneuronal excitability in healthy adults and post-stroke patients (Yi et al., 2017; He et al., 2019).

In the first part of the present study, to investigate the effects of acupoint acupuncture on LTP-like plasticity changes, we applied the intermittent theta-burst stimulation (iTBS) over the motor cortex of the upper extremities to induce LTP-like plasticity and then investigated the neuromodulation effect induced by acupuncture. In the second portion, we explored whether acupuncture could affect the acquisition of motor skills following repetitive exercise training.

## Materials and Methods

### Patients

A total of 37 healthy participants were recruited from Taihe Hospital in Shiyan City, and one withdrew due to illness. All healthy right-handed subjects had no history of head trauma, neurological disease, or other medical problems. Exclusion criteria included left-handedness, contraindications to TMS (e.g., metal residues, menstruating or pregnant women, etc.), taking any medication on a regular basis, and a positive history of psychiatric or neurologic diseases. Before the experiment, all participants were required to sign a written informed consent. The protocols were approved by the Ethics Committee of Taihe Hospital (Affiliated Hospital of the Hubei University of Medicine in China; approval number: 2014001-2).

### Experimental Design

**FIGURE 1:**
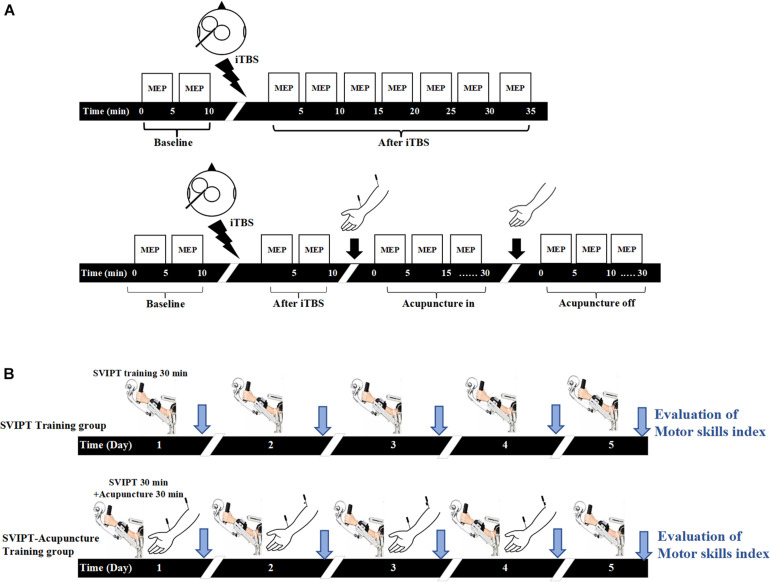
Experimental design. **(A)** In the intermittent theta-burst stimulation (iTBS) group, the baseline of motor evoked potential (MEP) amplitudes induced by transcranial magnetic stimulation (TMS) with the iTBS scheme over the primary motor cortex (M1) under resting conditions was measured for 10 min, then the LTP-like plasticity induced by iTBS was measured by TMS elicited MEP for 35 min. In the iTBS-acupuncture group, the changes of MEP were tested at pre- and post-iTBS for 10 min, then measured under acupuncture-in and off for 30 min after iTBS intervention. **(B)** Both groups carried on sequential visual isometric pinch task (SVIPT) training for 30 min, but the SVIPT-acupuncture group was given additional acupuncture after SVIPT training. After 10 min of SVIPT intervention, two needles were inserted into the Quchi and Waiguan acupoints and kept in the upper limb of the training side for 30 min and then extracted. The learning skill index was evaluated by video recorder continuously over 5 consecutive days.

The present study was divided into two parts (Figure 1). In the first experiment, the participants were randomly divided into iTBS (control group, *n* = 18) and iTBS-acupuncture (acupuncture group, *n* = 18) groups. The effects of TMS with the iTBS scheme on motor cortical excitability were verified (Figure 1A). Next, to observe the neuromodulation of aftereffects induced by the iTBS and the subsequent intervention of acupuncture, acupuncture was performed 10 min after iTBS, and the needle was *in-situ* for 30 min. The measurements of motor cortical excitability were assessed before iTBS and every 5 min for up to 70 min after the end of iTBS (Figure 1A).

In the second part (Figure 1B), to observe the effect of acupuncture on the acquisition of motor skills following repetitive exercise training, 36 participants were randomly divided into the sequential visual isometric pinch task (SVIPT) and SVIPT-acupuncture equal groups. The SVIPT training was conducted for 5 days on the left upper limb. The motor skill proficiency was assessed after each SVIPT training for five successive days. In the SVIPT-acupuncture group, with the same motor skill proficiency evaluation for five consecutive days, the SVIPT-acupuncture group received extra acupuncture with Quchi and Waiguan acupoints for 30 min on the same left upper limb after SVIPT repetitive training for four consecutive days. However, on the fifth day, the subjects only received the SVIPT training but not given the acupuncture. Under this experimental design, the immediate effect of acupuncture on the ability of motor skill acquisition can be observed for four successive days. In addition, with the avoidance of the immediate or short effect of acupuncture, the long-term effect of SVIPT combined with acupuncture on the acquisition of motor skills was assessed on the fifth day after 4 days of SVIPT-acupuncture intervention. All the above trial protocols have been registered with the China Clinical Trial Registry (registration no. ChiCTR-IPR-17010490).

### Assessment of Motor Cortical LTP-Like Plasticity

Motor cortical plasticity was evaluated by changes in MEP amplitude following iTBS. All TMS and TMS-iTBS interventions were performed using a transcranial magnetic stimulator (YRD-1, maximum magnetic field intensity = 2.2 T; Wuhan Yiruide Medical Equipment New Technology Co., Ltd., Wuhan, China) and a figure of eight coils (YRD, diameter = 9 cm; Wuhan Yiruide Medical Equipment New Technology Co., Ltd.). To determine the level of MEP amplitude, electromyographic (EMG) activity was obtained with disposable adhesive electrodes on the belly of the first dorsal interosseous (FDI) muscle and the metacarpophalangeal joint (Yahagi and Kasai, 1998). The EMG activity was amplified with (gain = 1000) and filtered using 50-Hz notches prior to digitization at 1 kHz (MP30, BIOPAC System, CA, United States).

To properly induce the MEP elicited by a single pulse of TMS, the center of TMS coils was focused on the right motor cortex corresponding to the left FDI muscle. The optimal scalp location, known as the hot spot, was determined by the scalp location at which MEPs could be elicited in the FDI muscle at the lowest stimulus strength. Once the hot spot was found, the coil was securely fixed in place with a mechanical device.

The resting motor threshold (rMT) was defined as the minimum stimulus intensity at which five of the 10 consecutive single stimuli at the hot spot elicited an MEP amplitude of at least 50 μV in the relaxed muscle (Rossini et al., 2015). The single pulse of the TMS intensity to elicit MEP during the entire stimulation paradigm was set at 120% rMT of the FDI. In the single iTBS group, the MEPs were measured “before” (10 min before iTBS, referred to as iTBS_*pre*_) and “after” (30 min after iTBS, referred to as iTBS_*post–*5 *min*_, iTBS_*post*–15 *min*_, and iTBS_*post*–30 *min*_). In the iTBS-acupuncture group, the MEP was measured “before” (10 min before iTBS, referred to iTBS’_*pre*_) and “after” (10 min after iTBS, referred to iTBS’_*post*–5_ and iTBS’_*post–*10_), as well as “in” (30 min with the needle *in situ*, referred to as acupuncture_*in–*5 *min*_, acupuncture_*in*–15 *min*_, and acupuncture_*in*–30 *min*_) and “off” (30 min after needle removal, referred to as acupuncture_*post–*5 *min*_, acupuncture_*post*–15 *min*_, and acupuncture_*post*–30 *min*_) (Figure 1A). The mean MEP amplitude was calculated as the average of the MEPs at each time point and then normalized to the 15-min pre-iTBS baseline.

### Intermittent Theta-Burst Stimulation and Acupuncture Intervention

To induce LTP-like plasticity, the intervention mode of iTBS was utilized in both groups over the right motor cortex corresponding to the left hand. The stimulus parameter of iTBS was a series of stimuli strings at 80% aMT of output intensity with a total of 600 TMS stimuli for 190 s. The iTBS protocol, containing 3-pulse bursts at 50 Hz repeated at 5 Hz, was performed with a 2-s train and repeated every 10 s for 20 repetitions (Franca et al., 2006; Hsieh et al., 2015). For acupuncture in-off, the iTBS-acupuncture group was under aseptic conditions and received extra acupuncture by experienced acupuncturists after 10 min of iTBS stimulation. The acupuncture needles were 0.3 × 40 mm (Huatuo; Suzhou Medical Supplies Factory, Suzhou City, China). The Quchi (LI-11) and Waiguan (TB-5) acupoints on the left hand are two of the most frequently explored points in Chinese acupuncture to treat poststroke motor dysfunction (Gao et al., 2011). To ensure that the patients felt De Qi (sensations of aching and tingling), proper techniques such as “lifting and thrusting” and no “rotating” were conducted at each point, and then the needles were kept *in situ* for 30 min without further stimulation.

### Motor Skills Training and Evaluation

**FIGURE 2:**
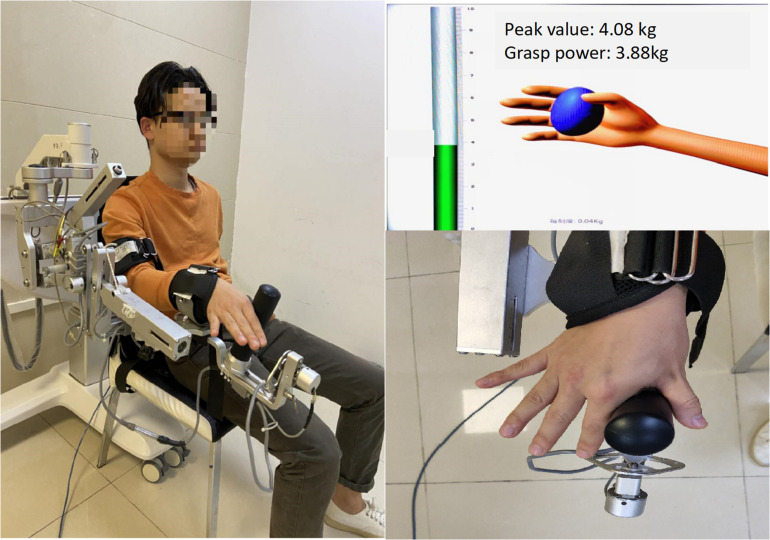
The SVIPT program. Participants were seated with the left shoulder and elbow flexed and the forearm in a neutral position and fixed on the upper limb exoskeleton of the intelligent feedback training system (IFTS). The radial side of the index finger and thumb was tightly attached to the columnar sensor. Participants learned to control column pressure to reach the target number range.

In the second part, the intelligent feedback training system (IFTS, SV3.8, Guangzhou Yikang Medical Equipment Industry Co., Ltd., Guangzhou, China) was used for motor skills training. For the starting position, participants were seated with the left shoulder and elbow flexed and the forearm in a neutral position and fixed on the upper limb exoskeleton of the IFTS. The wrist and finger joints were unrestricted, and the radial side of the index finger was tightly attached to the columnar sensor to receive pressure when the index finger was abducted. A 40-inch monitor was placed 1.5 m in front of the participants, where the height could be adjusted to the height of the participants’ eyes (Figure 2).

The motor skill proficiency was calculated using the SVIPT speed and error rates. Before implementing the SVIPT program, the maximum abducting force of each participant’s index finger was measured to ensure that the maximum pinching would not exceed the top of the column. The 0–50% range of the maximum abducting force of each participant’s index finger was marked on the pressure column and was divided into ten equal parts. The participants were able to control the pressure in the range of the marked number by abducting the index finger. Seven metronomes with different speeds (30, 45, 60, 75, 90, 105, and 120 bpm) were used to remind the participants to complete the action/movement/motion within each beat following the starting instructions and that at every beat, action/motion should be accomplished. Control of the cursor rose from 0 point to the corresponding number, and once it reached the target, the participant had control over the cursor to return to the 0 point as soon as possible. Participants repeated the above procedure in the order of 0–6–0–3–0–1–0–7–0–2–0–9–0–5–0–8–0–4–0. Finally, the cursor returned to the 0 point, with a total of nine target points in the order of: 6/3/1/7/2/9/5/8/4. Seven metronomes beats appeared randomly, and each beat was repeated three times with a 1 min rest between each task. We used a camera to monitor the entire process. Finally, the SVIPT-acupuncture group was given additional acupuncture by experienced acupuncturists. The needles’ Quchi and Waiguan acupoints were kept in the training side upper limb for 30 min and then extracted. Proper techniques such as “lifting and thrusting” and no “rotating” were conducted to make the patients experience De Qi. The acupuncture stimulus was repeated consecutively for 4 days, and no acupuncture was given after the fifth day of SVIPT training.

The hand performance of movement speed and errors were recorded by a video recorder continuously over five consecutive days after SVIPT training. SVIPT speed was the average time to complete three learning tasks in a row under each beat. Accuracy was defined as the number of times the cursor reached the serial number in three consecutive learnings at each metronome beat. The error rates were calculated as a 1 – accurate rate. The speed–accuracy tradeoff function (SAF) of each day quantified the acquisition ability of motor skills, calculated as the time of completion speed under each metronome beat as the abscissa and the corresponding error rate as the ordinate. The motor skills index was the change in SAF value using the following formula:

(1) $$\left(\mathrm{Skill}\,\mathrm{index},\text{SI}\right)=\frac{1-\mathrm{Error}\,\mathrm{rate}}{\mathrm{Error}\,\mathrm{rate}\,\left(\mathrm{In}\,{\left(\mathrm{Duration}\right)}^{b}\right)},b=5.424$$

### Statistical Analysis

STATA software was used to conduct statistical analyses (version 14.0; StataCorp, College Station, TX, United States). The MEP amplitude was calculated by the electromyogram software automatically. Three MEPs amplitudes every 5 min were measured to calculate the average amplitude and the average of the first three amplitudes (15 min) before the acupuncture were considered baseline. The average MEP amplitude in every 5 min divided by the baseline equaled the rate of change with the time as the abscissa and the change rate as the ordinate. The double factor variance analysis method was used to compare the changes in the skill index of the two groups over time. The *Q*-test was applied to intra-group and inter-group comparisons. The significance level was set at *P* < 0 05. Unless otherwise stated, values are reported as mean ± standard deviation.

## Results

### Motor Evoked Potential Amplitudes Were Consistently Higher After iTBS_post–30 *min*_ Stimulation

**FIGURE 3:**
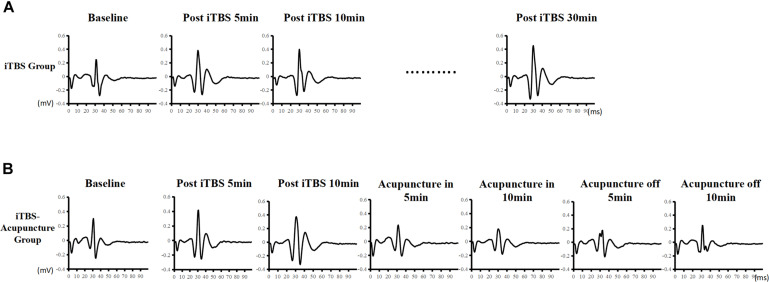
Effect of iTBS and acupuncture on MEPs. Time-course changes of MEPs following iTBS in panel **(A)** and iTBS and subsequent acupuncture intervention in panel **(B)**. Representative MEP traces following iTBS present an apparent increase, whereas there are traces of a decrease in MEP amplitude after subsequent acupuncture intervention.

**FIGURE 4:**
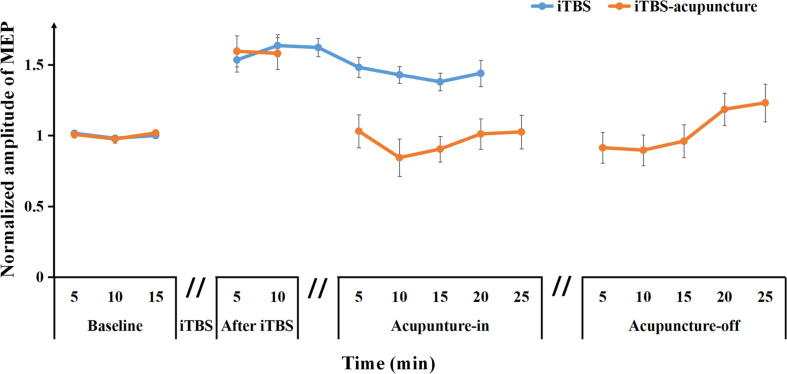
Averaged changes in the MEP amplitude at pre and after iTBS, as well as iTBS with subsequent acupuncture in-off intervention are presented. Data are shown as the mean ± SEM.

During iTBS intervention, subjects had no muscle contractions but felt scalp constrictions. The constrictions disappeared when the iTBS stimulation was stopped. The data showed that MEP amplitudes began to increase after iTBS stimulation. Representative changes in MEPs recorded at the iTBS_*pre*_ baseline, iTBS_*post–*5 *min*_, iTBS_*post*–15 *min*_, and iTBS_*post*–30 *min*_ are presented in Figure 3A. The MEP amplitudes after 10 min increased to 1.547 ± 0.454 mV, 1.5 times higher than the baseline value (*P <* 0.05). Next, MEP gradually decreased and flattened, but it was statistically higher than the baseline lasting for 30 min (*P* = 0.046). The MEP amplitude ratio after iTBS_*post*–30 *min*_ was 139 ± 16.89%, indicating that iTBS facilitated cortical excitability and induced LTP-like plasticity (Figures 3A, 4).

### Inhibition of MEP Amplitudes After iTBS-Acupuncture (In-Off) Stimulation

Representative changes in MEPs recorded at iTBS_*pre*_, iTBS_*post*–5_, iTBS_*post–*10_, acupuncture_*in*–5 *min*_, acupuncture_*in*–10 *min*_, acupuncture_*off*–5 *min*_, and acupuncture_*off*–10 *min*_ are presented in Figure 3B. Under acupuncture-in stimulation, the amplitude of MEP in a V-shaped change, as it first decreased and then increased, was always below the iTBS level (Figure 4).

After needling in the Quchi and Waiguan acupoints, the averaged normalized MEP amplitude decreased approximately to the baseline level (1.03 ± 0.34) during acupuncture_*in*–5 *min*_ and continued to drop to its lowest point (0.84 ± 0.39) during acupuncture_*in*–10 *min*_, and then began to increase to the baseline level during acupuncture_*in*–15–25 *min*_. During acupuncture-off stimulation, MEPs showed progressive enhancement for up to 25 min when compared with the baseline MEPs (Figure 4). This pattern demonstrates that acupuncture may suppress MEP amplitudes and disturb the maintenance of LTP-like plasticity induced by iTBS.

### Acupuncture Restrained the Learning Skill Index After Movement Practice

**FIGURE 5:**
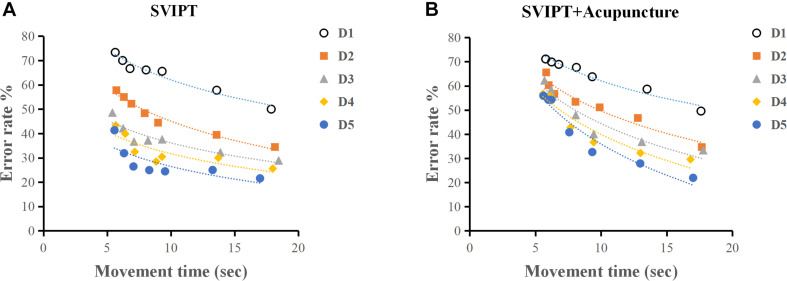
**(A)** As practice continued, participants in SVIPT groups obtained an apparent decrease in error rates with either fast or slow metronome velocity. **(B)** Acupuncture restrained the learning skill index after movement practice. The SVIPT-acupuncture group obtained higher error rates when the test speed was increased.

The SVIPT training effect was evaluated using the acquired learning skill index. A learning curve is defined as “the rate of skills or knowledge acquired over a period of time.” The learning curve consisted of the time abscissa of action completion and the error rate ordinate at each metronome velocity. The acquisition ability of motor skills after every day of training was assessed by SAF. As shown in Table 1 and Figure 5, both the SVIPT and SVIPT-acupuncture groups demonstrated that all participants had higher error rates while speed increased, especially during the first 2 days of training. As the training time increased, the subjects in the SVIPT group showed a noticeable decrease in error rates with either fast or slow metronome velocity. As seen in the SAF curve, when compared with the SVIPT group, the error rate in the SVIPT-acupuncture group was still maintained at a higher level with an increase in number of training days, especially at the higher speed (Figure 5).

**TABLE 1:** Learning skills index in sequential visual isometric pinch task (SVIPT) and SVIPT-acupuncture groups.

			120 bpm	105 bpm	90 bpm	75 bpm	60 bpm	45 bpm	30 bpm
SVIPT group	D1	Error rate	73.33	70.00	66.67	66.11	65.56	57.78	50.00
		Time	5.63	6.21	6.79	8.05	9.31	13.59	17.87
	D2	Error rate	57.78	55.00	52.22	48.33	44.44	39.44	34.44
		Time	5.72	6.31	6.90	7.94	8.99	13.58	18.16
	D3	Error rate	48.56	42.11	36.67	37.11	37.56	32.22	28.89
		Time	5.41	6.25	7.10	8.20	9.31	13.88	18.45
	D4	Error rate	43.33	39.89	32.44	30.44	28.44	30.00	25.56
		Time	5.67	6.42	7.16	9.30	8.84	13.72	17.99
	D5	Error rate	41.33	31.89	26.44	24.94	24.44	25.00	21.56
		Time	5.57	6.32	7.06	8.30	9.54	13.27	16.99
SVIPT-acupuncture group	D1	Error rate	71.11	69.89	68.89	67.64	63.78	58.65	49.56
		Time	5.78	6.23	6.78	8.12	9.33	13.50	17.61
	D2	Error rate	65.56	60.30	56.67	53.40	51.11	46.70	34.67
		Time	5.82	6.01	6.44	8.05	9.89	12.80	17.67
	D3	Error rate	62.22	58.40	55.56	47.90	40.00	36.80	33.33
		Time	5.69	6.20	6.30	8.07	9.44	13.10	17.78
	D4	Error rate	56.67	55.80	55.56	42.80	36.67	32.20	29.56
		Time	5.58	6.15	6.00	7.68	9.44	13.00	16.81
	D5	Error rate	55.89	54.21	54.28	40.79	32.60	27.87	21.93
		Time	5.61	6.20	5.98	7.58	9.33	12.98	17.01

The learning skill index in the SVIPT group was higher than that of the SVIPT-acupuncture group. The result of two-factor ANOVA showed a statistically significant difference between the two groups (*F* = 50.68, *P* < 0.01). The learning skill indices had a cumulative effect on time and training. Alternatively, with the accumulation of time and quantity of training, the participants engaged in SVIPT motor practice without acupuncture demonstrated a better average learning skill index than participants with additional acupuncture (Table 1). No significant differences were found in the first 2 practice days (*q* = 0.001, *P* = 0.68; *q* = 0.022, *P* = 0.743). Over time, the SVIPT group demonstrated a progressively higher index than the SVIPT-acupuncture group on the third (*q* = 0.035, *P* = 0.058), fourth (*q* = 0.083, *P* < 0.01), and fifth days (*q* = 0.088, *P* < 0.01) (Table 2). These results suggest that acupuncture intervention may restrain the subjects’ learning skill index after movement practice and could inhibit the cortical plasticity induced by the SVIPT practice.

**TABLE 2:** Learning skill index for 5 days in the SVIPT and SVIPT-acupuncture groups.

	SVIPT group	SVIPT-acupuncture group	*q*	*P*
D1	0.082 ± 0.013	0.083 ± 0.014^#^	0.00104	0.68
D2	0.107 ± 0.015	0.090 ± 0.022^#^	0.02202	0.743
D3	0.189 ± 0.019	0.098 ± 0.022^#^	0.03545	0.058
D4	0.187 ± 0.016	0.104 ± 0.020**	0.08276	<0.01
D5	0.213 ± 0.019	0.126 ± 0.027**	0.08777	<0.01

^#^Represents *P* > 0.05. **Represents *P* < 0.01.

## Discussion

Several notable results were demonstrated in the present study. First, iTBS facilitated cortical excitability and induced LTP-like plasticity over the M1 cortex. Second, unilateral acupuncture in off stimulation reversed the maintenance of LTP-like plasticity induced by iTBS. Third, these results suggested that subsequent acupuncture intervention after movement practice may inhibit learning skill indices following repetitive exercise training, indicating that subsequent acupuncture in the contralateral upper limb had a negative impact on the behavioral outcomes of motor learning.

In the present study, we measured cortical plasticity as reflected by MEP changes. iTBS over the M1 region was proven to initiate a long-term increase in MEP amplitude that was higher than the baseline (139 ± 16.89%, *P* = 0.046) and LTP-like plasticity had a duration longer than 30 min. This is consistent with the literature and it is believed that LTP-like plasticity is produced over the stimulus cortical side. Preclinical and clinical studies have confirmed that the induction of LTP is related to the intensity and time-dependent on stimuli (Fino et al., 2010; Zhang et al., 2010), regulates the magnitude and direction of MEP induction (He et al., 2019), and is involved in two mechanisms of homeostatic plasticity or gating mechanisms (Karabanov et al., 2015; Yee et al., 2017). Recently, several studies have reported that homeostatic plasticity plays an important role in the control of synaptic plasticity. In accord with the Bienenstock–Cooper–Munro (BCM) rule contribute to regulating plasticity (Bienenstock et al., 1982), the plasticity induced by motor skill training, transcranial direct current stimulation (tDCS) or paired associative stimulation (PAS) changed the synaptic modification threshold or regulated excitability in the motor cortex (Rioult-Pedotti et al., 2000; Siebner et al., 2004). In the current study, the changes of LTP-like plasticity induced by iTBS or acupuncture could be expected from a homeostatic mechanism that controls the motor cortical excitability. Gating mechanisms could also be involved in the changes of LTP-like plasticity induced by iTBS or acupuncture. The gating mechanisms, including transiently suppressing the efficacy of intracortical inhibitory circuits, shifting intrinsic excitability of the targeted neurons or increasing net calcium influx into the targeted cortical neurons, may promote the induction of LTP-like plasticity in neural circuits targeted by iTBS, physical training or acupuncture (Ziemann and Siebner, 2008; Karabanov et al., 2015).

The present study concluded that acupuncture at the Quchi and Waiguan acupoints reversed the LTP induced by iTBS, indicating that high MEP gradually reduced to the baseline level after acupuncture-in and did not return to the iTBS level after needle extraction. This suggests a time-dependent reversal of LTP by unilateral acupuncture in-off stimulation, considered as “depotentiation” (Huang and Hsu, 2011). Concurrent with previous studies (Kramar and Lynch, 2003), subsequent low-frequency afferent stimulations (LFS), such as brief periods of hypoxia, application of receptor, and brief cooling shock reversal of the depotentiation (Kulla and Manahanvaughan, 2000; Huang and Hsu, 2011; Park et al., 2019), were introduced after TBS completely reversed LTP. As we adopted acupoint acupuncture as the subsequent spike, in the phase of acupuncture-in, the MEP amplitude demonstrated a V-shaped change, as it decreased after acupuncture_*in*–5 *min*_ administration, until it reached the lowest point (0.90 ± 0.25) after acupuncture_*in*–15 *min*_, and then slowly recovered to the equivalent baseline level (1.01 ± 0.36) after acupuncture_*in*–30 *min*_. In acupuncture-off, the MEP continued to reach the lowest point (0.91 ± 0.42) after acupuncture_*off–*5 *min*_, and slowly increased to approximately 1.18 times of the baseline (1.18 ± 0.40) after acupuncture_*off*–20 *min*_. These results indicated that the depotentiation effects of the motor cortex-corticospinal tract comprised a reversal of iTBS induced LTP, which occurred following acupuncture. This potentiation may provide a mechanism for preventing the saturation of synaptic potentiation and increase the efficiency and capacity of the information storage of the neuronal network (Kulla and Manahanvaughan, 2000; Chen et al., 2001; Huang and Hsu, 2011; Park et al., 2019).

The mechanisms responsible for the depotentiation effect of acupuncture following LTP induction are unclear, and metaplasticity does not explain the results. Our previous study demonstrated that the MEP amplitude after simple acupuncture-in gradually decreased and slowly recovered in the shape of a “V,” immediately rose, and then continued to slowly rise after extraction of the needle. Both simple acupuncture processes did not exceed 20 min, which may be a short-term inhibition or potentiation, in accordance with the results of simple acupuncture examinations of the modulatory effect on cortical plasticity (Chen et al., 2006; He et al., 2019). Researchers have suggested that simple acupuncture needling or LFS (Huang et al., 2001) that affects cortical excitability may be related to the plasticity processes mediated by *N*-methyl-D-aspartic (NMDA) receptors. To a certain extent, we suggest that the functional state of GABA_*b*_ receptors involved in acupuncture may be partially interpreted as decreased cortex excitability during the acupuncture-in state reversed by iTBS induction. However, the mechanisms of decreased cortex excitability during the acupuncture-off state persistently reversed iTBS induction, although this was unclear due to the increased MEP after needle extraction. Some researchers have also found that LTP stabilization requires protein synthesis in the consolidation of long-term memory (Abraham and Williams, 2008), and that the pharmacological inhibition of proteasome-dependent protein degradation would disrupt the expression of late (L-) LTP (Fonseca et al., 2006). Researchers have also emphasized that brain-derived neurotrophic factors (BDNFs) can sustain L-LTP through PKM when protein synthesis is inhibited fluence the subsequent induction ability of the latter stimulus (Abraham, 2008). Depotentiation effects have shown that synaptic modifications can be reversed by subsequent stimuli, which inhibit the maintenance of LTP instead of reversing LTP into LTD; however, these effects do not demonstrate a long-term change. This finding has been demonstrated previously, as LTP of hippocampal synapses in adult rats was reversed as the rats entered a novel environment (Zhou and Poo, 2004; Abraham, 2008). Previous studies have revealed that acupuncture mainly comes with short-term potentiation/depression and that it modulates an opposite effect on MEP change with slight depression after needling-in and significant facilitation after needling-off (Zhou and Poo, 2004; He et al., 2019). We found that iTBS-induced LTP-like plasticity can be inhibited by either needle in or needle off, which indicates that depotentiation, rather than metaplasticity, occurs in motor cortical circuits; however, little is known about its molecular mechanisms.

Recently, considerable interest has been generated by the possibility that the activity-dependent persistent reversal of previously established synaptic LTP (depotentiation) may be important in the time- and state-dependent erasure of memory. We used the experimental scheme of SVIPT (Abraham, 2008; Reis et al., 2009) and acupuncture to explore whether SVIPT-acupuncture can affect the acquisition ability of learning skills. The SAF curve showed that the SVIPT group without acupuncture demonstrated a more satisfactory performance than the SVIPT-acupuncture group with an acupoint needle, especially as practice time continued, in reducing error rates with either the fast or slow metronome. Simultaneously, the learning skill index in the SVIPT group was significantly higher than that of the SVIPT-acupuncture group (*F* = 50.68, *P* < 0.01). These results suggest that subsequent acupoint acupuncture after motor skill training inhibited the maintenance of LTP-like plasticity and restrained the subjects’ learning ability, which was consistent with this electrophysiological results. The present and previous studies are concordant because they both demonstrated that abnormal maintenance of LTP/LTD would lead to difficulty in the reconstruction of the normal motor in learning ability in preclinical Parkinson’s models (Picconi et al., 2003). Exercise training is closely related to cortical motor regions, and how depotentiation affects motor skill acquisition needs further research. This behavioral data showed that the learning skill indices in the SVIPT-acupuncture group were lower than those of the SVIPT group, but a small amount of skill improvement could still be achieved through daily training. The underlying reasons for this difference require further analysis.

In this study, we first observed the effects of acupuncture on the left upper limb area corresponding to contralateral motor cortical excitability. In the secondary objective, we further investigated the interaction between acupuncture and the acquisition of learned skills. In addition, many existing studies on the interaction of non-invasive brain stimulation technologies such as repetitive TMS and transcranial direct current stimulation have verified synaptic metaplasticity (Hamada et al., 2008; Pötter-Nerger et al., 2009), while this research was focused on depotentiation. We propose that subsequent acupuncture may negatively affect the efficacy of learned skills after iTBS or rehabilitation training.

## Conclusion

The repetitive TMS Evidence-based guidelines recommend the use of HF-rTMS or iTBS over the M1 area on the affected side, which can facilitate the M1 excitability and LTP effect and improve recovery of upper limb function in stroke patients (Lefaucheur et al., 2020). Acupuncture can specifically modulate the excitability of the contralateral primary motor cortex (M1) (Ferbert et al., 1992). However, these two different therapies were dependent on the therapist’s schedule in clinical practice. It has not been reported whether the order of application of iTBS and acupuncture affects the therapeutic effect and the mechanism is unclear. This research attempts to explore this relationship and contribute to this regard. The results of the present study indicate that unilateral acupuncture in-off can reverse LTP-like plasticity of the contralateral motor cortex induced by iTBS, namely acupuncture electrically induced synaptic depotentiation. Subsequent continuous acupuncture on the upper limbs of the training side may inhibit the acquisition of learned skills during repetitive exercise training. Understanding the neurophysiological or cellular basis of motor cortical plasticity may help gain insight into the long-term synaptic plasticity involved in rehabilitation therapy and neuromodulatory technologies (such as iTBS and acupuncture), thereby revealing potential therapeutic targets for neurological diseases.

## Data Availability Statement

The raw data supporting the conclusion of this article will be made available by the authors, without undue reservation.

## Ethics Statement

The trial protocols have been registered with the China Clinical Trial Registry (Registration No. ChiCTR-IPR-17010490). The protocols were approved by the Ethics Committee of Taihe Hospital (Affiliated Hospital of Hubei University of Medicine in China; Approval Number: 2014001-2). The patients/participants provided their written informed consent to participate in this study.

## Author Contributions

X-KH and H-HL designed the study. LL, GY, and Z-YN conducted the study, including patient recruitment and data collection. X-KH and L-DC contributed to data analysis. H-HL and S-JC prepared the manuscript draft with important intellectual input from X-KH and T-HH. All authors approved the final manuscript. All authors listed have made a substantial, direct and intellectual contribution to the work, and approved it for publication.

## Conflict of Interest

The authors declare that the research was conducted in the absence of any commercial or financial relationships that could be construed as a potential conflict of interest.
